# Chlorella-Induced Increase in Cardiac Function Further Enhances Aerobic Capacity Through High-Intensity Intermittent Training in Healthy Young Men and Rats

**DOI:** 10.3390/nu17162657

**Published:** 2025-08-16

**Authors:** Shumpei Fujie, Kenichiro Inoue, Katsunori Tsuji, Naoki Horii, Moe Oshiden, Izumi Tabata, Motoyuki Iemitsu

**Affiliations:** 1 Faculty of Sport and Health Science, Ritsumeikan University, Kusatsu 525-8577, Japan; s-fujie@fc.ritsumei.ac.jp (S.F.); sh0118es@ed.ritsumei.ac.jp (K.I.); katsuj@ncc.go.jp (K.T.); 819mooesoo@gmail.com (M.O.); tabatai@fc.ritsumei.ac.jp (I.T.); 2 Sports Research Center, Hosei University, Kawasaki 211-0031, Japan; 3 Japan Society for the Promotion of Science, Tokyo 102-0083, Japan; naoki352c@gmail.com

**Keywords:** maximal oxygen uptake, microalgae, exercise, stroke volume

## Abstract

Background: Chronic chlorella intake combined with high-intensity intermittent training (HIIT) has been shown to accelerate aerobic and anaerobic capacities in rodents. This study aimed to clarify the effects of combining chlorella intake with short-term HIIT on exercise performance in humans, and to investigate the impact of chlorella intake on cardiac adaptation. Materials and Methods: In Study 1, twelve healthy young men completed a 3-week exhaustive HIIT, comprising 6–7 bouts of 20 s of cycling on a leg ergometer at an intensity of 170% maximal oxygen uptake ($\dot{V}$O_2max_), with a 10 s rest between each bout, 3 days/week. They were orally administered either placebo or chlorella during the 3 weeks in a double-blinded, randomized crossover trial (RCT). In Study 2, six healthy young men were orally administered either placebo or chlorella during 4 weeks in a double-blinded, placebo-controlled RCT. We measured $\dot{V}$O_2max_ and cardiac function (stroke volume [SV], heart rate [HR], and cardiac output [CO]) during maximal exercise. In Study 3, chlorella-induced changes in molecular markers of maladaptation of the heart were measured in healthy rats. Results: [Study 1] After each HIIT, $\dot{V}$O_2max_ significantly increased in the placebo and chlorella groups (*p* < 0.05). Changes in $\dot{V}$O_2max_ were significantly higher in the chlorella group than in the placebo group (*p* < 0.05). [Study 2] Changes in SV and CO during maximal exercise were significantly higher in the chlorella group than in the placebo group (*p* < 0.05 each), but HR_max_ did not change. [Study 3] Chronic chlorella intake did not change the molecular markers of pathological cardiac hypertrophy. Conclusions: Chronic chlorella intake, which improves aerobic capacity by enhancing cardiac function without causing cardiac maladaptation, combined with short-term HIIT, further enhanced aerobic capacity. Thus, the chlorella-induced increase in cardiac function may further enhance aerobic capacity through HIIT.

## 1. Introduction

High-intensity intermittent training (HIIT) is characterized by brief, repeated bursts of intense exercise separated by periods of rest, and it is an effective tool for increasing exercise performance in terms of both maximal oxygen uptake ($\dot{V}$O_2max_) as an index of aerobic capacity and maximal accumulated oxygen deficit (MAOD) as an index of anaerobic capacity [1,2]. Additionally, short-duration HIIT—total session duration ≤ 30 min (including warm-up, recovery, and cool-down)—is a time-efficient exercise strategy [1]. Chronic short-duration HIIT is well studied, and the evidence remains consistent with respect to its positive impact on whole-body physiological parameters, including increases in both aerobic and anaerobic capacities in untrained adults, active adults, and elite athletes [3,4,5,6,7]. With growing interest in optimizing training outcomes, exploring nutritional strategies to further enhance HIIT-induced exercise capacity is essential.

Chlorella is a unicellular green alga that contains a variety of proteins, minerals, vitamins, and amino acids and is used as a multi-component supplement [8]. Chronic chlorella intake prevents obesity, diabetes, and cardiovascular disease, while also accelerating immune function [8,9,10,11,12]; a positive effect on exercise performance has also been observed. Chlorella supplementation for 4 weeks elevates peak oxygen uptake in young men [13]. Moreover, chlorella supplementation for 2 weeks significantly extended maximal swimming times in mice [14]. Our previous study showed that chlorella supplementation for 6 weeks increased the maximal repetition number of high-intensity intermittent exercise (HIIE), which is composed of 20 s swimming sessions while bearing a weight equivalent to 16% of their body weight, with a 10 s rest period between sessions, in rats [15]. Furthermore, the combination of chlorella intake and HIIT intervention additively increased the maximal repetition number of HIIE compared with each alone, along with additively activated muscle oxidative and glycolytic metabolism [15]. Conversely, in women with overweight and obesity, an 8-week chlorella intake combined with HIIT intervention did not change $\dot{V}$O_2max_ [16]. However, it is unclear whether chronic chlorella intake combined with HIIT has a more pronounced effect on exercise performance because of the uncertainty in translating these findings from rodents to humans.

Herein, we hypothesized that combining HIIT and chlorella intake intervention would increase exercise performance, including aerobic and anaerobic capacities, compared with HIIT alone. To test this hypothesis, we investigated their combined effects on $\dot{V}$O_2max_ and MAOD (Study 1). Aerobic capacity is influenced by both maximal cardiac output (CO) [17] and muscle oxidative capacity [18]. Although our previous study demonstrated that chlorella intake increased muscle oxidative metabolic capacity, its effect on maximal CO remains unclear. Therefore, we hypothesized that chlorella intake enhances CO during maximal exercise through greater left ventricular (LV) filling and contractility. To understand the mechanism underlying the effect of chlorella intake on aerobic capacity, we examined cardiac function and morphology at rest and during maximal exercise before and after chlorella intake (Study 2). Furthermore, based on the results of Study 2, it remains unclear whether the cardiac adaptations associated with the chlorella-induced increase in $\dot{V}$O_2max_ are adaptive or maladaptive. Thus, we investigated whether chlorella alters the expression of cardiac maladaptive molecular markers (Study 3).

## 2. Materials and Methods

### 2.1. Study 1

#### 2.1.1. Participants

Twelve healthy young male volunteers (age, 21 ± 1 years; height, 174 ± 7 cm) participated in this double-blinded, randomized, placebo-controlled, crossover study with a 4-week washout period; using computer-generated random numbers, they were randomly divided into two groups: the HIIT combined with placebo intake (HIIT + PL) group and the HIIT combined with chlorella intake (HIIT + CH) group. The exclusion criteria were as follows: (1) individuals taking medications or supplements, (2) individuals with a history or current diagnosis of cardiovascular disease or musculoskeletal injuries, and (3) individuals who exercised regularly. Exclusion and inclusion criteria were evaluated via questionnaires and medical history interviews. Participants who fulfilled the eligibility criteria were enrolled in this study. When the participation rate in training sessions and/or chlorella intake was 90% or less, the exclusion criteria were applied due to “low adherence to the exercise intervention and supplement intake intervention.” However, the participation rate in this study was 100% (i.e., no adverse events or participant dropouts were observed). The participants had a sedentary lifestyle and did not participate in vigorous sports activities. They were excluded if they were taking any medications or supplements or had a history of chronic diseases. They barely consumed alcohol, and none had a history of smoking for at least 12 months prior to the study. No significant differences in total energy intake or macronutrient composition were observed before and after the intervention period as assessed using the validated brief, self-administered diet history questionnaire (BDHQ), which includes 73 items. All participants were informed of the experimental procedures and risks and provided written informed consent before participating in the study. The study was approved by the Ethics Committee of Ritsumeikan University (BKC-2015-010) and conducted in accordance with the Declaration of Helsinki. The study was registered in the University Hospital Medical Information Network Clinical Trials Registry (UMIN-CTR; UMIN000054262).

#### 2.1.2. Experimental Design

All the volunteers participated in the HIIT + PL and HIIT + CH groups in a randomized order. Twelve healthy young men completed a 3-week exhaustive HIIT, comprising 6–7 bouts of 20 s of cycling on a leg ergometer at an intensity of 170% $\dot{V}$O_2max_ with a 10 s rest between each bout [2], 3 days/week. The $\dot{V}$O_2max_ was measured approximately 1 week before each intervention. The participants orally consumed chlorella (Sun Chlorella Corp., Kyoto, Japan) or a placebo (15 tablets) after breakfast and dinner during the 3-week period in this double-blinded, randomized, crossover study with a 4-week washout period. Measurements were taken before and after the 3-week participation period in both groups, including body weight, body fat mass, lean body mass, resting heart rate (HR), systolic blood pressure (SBP), diastolic blood pressure (DBP), $\dot{V}$O_2max_, and MAOD. Body weight, body fat mass, and lean body mass were assessed using an InBody 770 body composition analyzer (Biospace, Tokyo, Japan). The resting HR, SBP, and DBP were assessed using an HBP-9020 automated oscillometric device (Omron Healthcare, Kyoto, Japan). Measurements after the intervention were performed at least 48 h after the last HIIE session to avoid the influence of acute effects of exercise. All measurements were performed at a constant room temperature (24 ± 1 °C). All assessments, associated evaluations, and data analyses were blinded to ensure objectivity and minimize potential bias.

#### 2.1.3. Chlorella and Placebo Tablets

The chlorella and placebo tablets used in this study were the same as those used in a previous study [13]. The main ingredient of the chlorella tablets was dried *Chlorella pyrenoidosa* powder, and the main components of the placebo tablets were lactose and colorant. The color and shape of both tablets were indistinguishable and identical. The placebo tablets were produced using a specific process: a mixture of lactose (82.5%) and colorant (17.5%) was dissolved in purified water, dried, and blended with sucrose fatty acid ester (5%). The final mixture was then compressed into tablet form [12]. In contrast, the chlorella tablets were primarily composed of dried *Chlorella pyrenoidosa* powder as the active ingredient. The participants were randomized to receive either the chlorella or placebo in a double-blinded manner. Chlorella and placebo tablets were visually identical and coded by a third-party researcher who was not involved in data collection or analysis. Allocation codes were securely stored and only revealed after data analysis was completed, ensuring that both participants and investigators remained blinded throughout the study. During each trial, participants consumed 30 tablets per day (15 tablets twice daily after breakfast and dinner) [13]. No participant forgot to take a tablet throughout the 3-week intervention.

#### 2.1.4. Measurement of $\dot{V}$O_2max_

To determine the linear relationship between exercise intensity and steady-state oxygen uptake using the Douglas-bag method, each participant performed six to eight 10 min exercises at constant power (between 35% and 90% $\dot{V}$O_2max_), using intermittent multi-stage incremental load methods as described in a previous study [2]. All exercises were performed at a pedaling frequency of 90 rpm. The oxygen and carbon dioxide fractions in the expired air were measured using a mass spectrometer (ARCO-2000; ARCO System, Chiba, Japan). The gas volume was measured using a dry gas meter (DC-2; Shinagawa Seisakusho, Tokyo, Japan). After confirming that leveling-off of oxygen uptake occurred with increasing intensity, the highest VO_2_ among the trials was designated as $\dot{V}$O_2max_ [19]. To enhance reproducibility, $\dot{V}$O_2max_ was measured multiple times per participant in this study, yielding coefficients of variation (CV) of 0.027 ± 0.049%.

#### 2.1.5. Measurement of MAOD

MAOD was determined as an index of anaerobic capacity during a 2–3 min exhaustive bicycle exercise using the Douglas-bag method, as described in previous studies [2]. The exercise intensity was chosen individually for each participant to cause exhaustion within 2–3 min time frames. The accumulated oxygen demand was calculated as the product of the estimated oxygen demand and exercise duration. The accumulated oxygen deficit was calculated as the difference between the accumulated oxygen demand and the accumulated oxygen uptake.

#### 2.1.6. HIIT Intervention

All the participants completed short-lasting, exhaustive 3-week HIIT (100% compliance), 3 days/week under the supervision of an instructor. Each session consisted of 6–7 sets of 20 s of cycling on a leg ergometer (828E, Monark, Vansbro, Sweden) at an intensity of 170% of $\dot{V}$O_2max_ at 90 rpm, with a 10 s rest between each bout according to a previous study [2]. A linear extrapolation to higher power was performed to determine 170% $\dot{V}$O_2max_ and the corresponding bicycling exercise intensity using the established relationship between power and steady-state oxygen uptake, as described above. When participants completed more than seven exercise sets, the exercise intensity was increased by 11 W. Additionally, to account for participant fatigue, training sessions were scheduled with at least 1 day of rest between sessions, rather than on consecutive days.

### 2.2. Study 2

#### 2.2.1. Participants

Six healthy young male volunteers (age, 22 ± 2 years; height, 178 ± 6 cm) participated in this double-blinded, randomized, placebo-controlled, crossover study with a 4-week washout period. They were randomly divided into two groups: the placebo intake (PL) group and the chlorella intake (CH) group. The participants in Study 2 were independent from those of Study 1. The exclusion and inclusion criteria, as well as the screening procedures, were identical to those used in Study 1. Based on the screening results, all participants were healthy individuals with no regular exercise habits, no supplement or medication use, and no history of chronic diseases. The participants had a sedentary lifestyle and did not participate in vigorous sports activities. The criteria for excluding participants were the same as in Study 1. No significant differences in total energy intake or macronutrient composition were observed before and after the intervention period, as assessed using the validated BDHQ, which includes 73 items. All participants were informed of the experimental procedures and risks and provided written informed consent before participating in the study. The study was approved by the Ethics Committee of Ritsumeikan University (BKC-2019-049) and conducted in accordance with the Declaration of Helsinki. The study was registered in the University Hospital Medical Information Network Clinical Trials Registry (UMIN-CTR; UMIN000054263).

#### 2.2.2. Experimental Design

All participants were assigned to the PL and CH groups in a randomized order, using computer-generated random numbers. They orally consumed either a placebo or chlorella after breakfast and dinner for 4 weeks in this double-blinded, randomized, crossover study with a 4-week washout period. When the participation rate in chlorella intake was 90% or less, the exclusion criteria were applied due to “low adherence to the supplement intake intervention.” However, the participation rate in this study was 100% (i.e., no adverse events or participant dropouts were observed). Measurements were performed before and after the 4-week participation period in both groups and included body weight, body fat mass, lean body mass, and $\dot{V}$O_2max_. To clarify the mechanism of the effect of chlorella intake on aerobic capacity, we measured HR, stroke volume (SV), CO, ejection fraction (EF), fractional shortening (FS), left ventricular end-systolic diameter (LVESD), left ventricular end-diastolic diameter (LVEDD), interventricular septal thickness (IVST), left ventricular posterior wall thickness (LVPWT), and LV mass index at rest and/or during maximal exercise (measurement of $\dot{V}$O_2max_) as indicators of cardiac function and morphology. All measurements were performed at a constant room temperature (24 ± 1 °C). All assessments, associated evaluations, and data analyses were conducted in a blinded manner to ensure objectivity and minimize potential bias. Additionally, the measurements and analyses of echocardiographic or $\dot{V}$O_2max_ were performed on different individuals.

#### 2.2.3. Chlorella and Placebo Tablets

The chlorella and placebo tablets used in this study were the same as those used in Study 1 of this study and a previous study [13]. The participants were randomized to receive either a placebo or chlorella in a double-blinded manner. No participant forgot to take the tablet throughout the 4-week intervention.

#### 2.2.4. Measurement of $\dot{V}$O_2max_

$\dot{V}$O_2max_ was measured using the same methods and equipment as in Study 1 of this study. To enhance reproducibility, $\dot{V}$O_2max_ was measured multiple times per participant in this study, yielding CV of 0.032 ± 0.061%.

#### 2.2.5. Measurements of Cardiac Function and Morphology

At rest and during maximal exercise, SV, EF, FS, LVESD, LVEDD, IVST, and LVPWT were measured in the sitting position on a leg ergometer using an ultrasound system (Vivid S6, GE Healthcare Japan, Tokyo, Japan), as described in previous study [20]. The cross-sectional area (CSA) of the ascending aorta was determined by measuring the diameter at the point where the aortic valve leaflets meet using M-mode echocardiography on the left sternal border of the 4th rib and was calculated using the average of the largest aortic diameters in systole and end diastole. Simultaneously, LVESD, LVEDD, IVST, and LVPWT were measured, and then EF and FS were calculated. The LV mass index was calculated using the formula: LV mass = 1.04 ([LVEDD + LVPWT + IVST]^3^-LVEDD^3^) × 0.8 + 0.6 [21]. Doppler echocardiography was used to measure the blood flow velocity of the ascending aorta and was performed from the suprasternal notch using a 2.5-MHz Doppler transducer (Vivid S6, GE Healthcare). SV was calculated as the product of the CSA of the ascending aorta and blood flow during a cardiac cycle [20,22]. The average of a minimum of 10 cardiac cycles were used to calculate SV. CO was calculated as the product of SV and HR.

### 2.3. Study 3

#### 2.3.1. Animals and Protocol

Ethical approval for this study was obtained from the Committee on Animal Care at Ritsumeikan University (BKC-2013-010). Twelve-week-old male Sprague–Dawley rats were obtained (CLEA Japan, Tokyo, Japan) and cared for according to the Guiding Principles for the Care and Use of Animals based on the Declaration of Helsinki. Neither animals nor data points were excluded from the experiments and data analysis, and predefined criteria were not used. The rats were housed individually in an animal facility under controlled conditions (a 12:12 h light–dark cycle). Using computer-generated random numbers, they were randomly divided into two groups (*n* = 10): a control (CON) group and a chlorella intake (CH) group. The sample size of experimental units was carefully determined to minimize pain and distress, in strict adherence to the principles of replacement, reduction, and refinement, as recommended by the committee. The CON group was provided access to water and fed normal chow (CE-2; CLEA Japan) ad libitum during the 6-week period. The CH group was fed chow containing 0.5% chlorella powder. Chow was removed from the cages of all rats 12 h before harvesting the heart to avoid the acute effects of chlorella intake. As described in a previous study [23], HR, SBP, and DBP were measured under anesthesia. The heart was resected quickly, rinsed in ice-cold saline, weighed, and frozen in liquid nitrogen. Anatomical and biochemical experiments, as well as their analyses, were conducted in a blinded manner.

#### 2.3.2. Real-Time RT-PCR

Total tissue RNA (20 ng) in heart samples was isolated using the ReliaPrep RNA Tissue Miniprep System (Promega, Madison, WI, USA), along with the RNeasy mini kit (#74104, Qiagen, Hilden, Germany), with reference to a previous study [24]. Single-stranded cDNA was synthesized from prepared RNA using OmniScript reverse transcriptase (Qiagen). The mRNA expressions of atrial natriuretic peptide (ANP; assay ID, Rn00664637_g1; Applied Biosystems, Foster City, CA, USA), endothelin-1 (ET-1; assay ID, Rn00561129_m1; Applied Biosystems), and angiotensin-converting enzyme (ACE; assay ID, Rn00561094_m1; Applied Biosystems) in the heart were used as molecular markers of maladaptation and analyzed using real-time reverse transcription polymerase chain reaction (RT-PCR) with TaqMan Gene Expression assays. Real-time RT-PCR was performed on a Prism 7500 Fast Sequence Detection System 2.2 (Applied Biosystems), and cycle threshold values were calculated using the system’s software. ANP, ET-1, and ACE mRNA expression levels were normalized against the expression of β-actin mRNA in the same sample.

#### 2.3.3. Immunoblot

Western blot was performed to detect heart phosphorylated extracellular signal-regulated kinase (ERK1/2; p44/p42), total ERK1/2, phosphorylated Jun-N-terminal kinase (JNK1/2; p54/p46), total JNK, phosphorylated mitogen-activated protein kinase p38 (p38), and total p38 in both nuclear and cytosolic fractions as molecular markers of maladaptation of the heart. We homogenized the heart tissue sample and separated nuclear and cytosolic fractions. Briefly, proteins from heart tissue homogenates, including all fractions (40 µg of total protein), were separated using 10% sodium dodecyl sulfate–polyacrylamide gel electrophoresis and transferred to polyvinylidene fluoride membranes (Millipore, Billerica, MA, USA). The membranes were incubated for 1 h in a blocking buffer (2% skim milk in phosphate-buffered saline [PBS] with 0.1% Tween 20 [PBS-T]) and for 12 h with antibodies against phosphorylated ERK1/2 (1:1000; Cell Signaling Technology, Tokyo, Japan), total ERK1/2 (1:1000; Cell Signaling Technology), phosphorylated JNK1/2 (1:1750; Cell Signaling Technology), total JNK (1:1000; Cell Signaling Technology), phosphorylated p38 (1:750; Cell Signaling Technology), and total p38 (1:1000; Cell Signaling Technology) in a blocking buffer at 4 °C. The membranes were rinsed thrice with PBS-T and incubated for 1 h at room temperature (24 °C) with horseradish peroxidase–conjugated secondary anti-rabbit or anti-mouse (1:2000) immunoglobulins in a blocking buffer. After rinsing the membranes with PBS-T, phosphorylated ERK1/2, total ERK1/2, phosphorylated JNK1/2, total JNK, phosphorylated p38, and total p38 were detected using the Enhanced Chemiluminescence Plus system (GE Healthcare Bio-Sciences AB, Uppsala, Sweden) and visualized on a FUSION FX machine (Vilber Lourmat, Collégien, France).

### 2.4. Statistical Analysis

In Study 1, values are expressed as mean ± standard deviation (SD). An unpaired *t*-test was used to assess differences at baseline and changes in each parameter before and after each intervention between the HIIT + PL and HIIT + CH groups in Study 1. Differences before and after each intervention were compared using a paired *t*-test.

In Study 2, values are expressed as mean ± SD. An unpaired *t*-test was used to assess differences at baseline and changes in each parameter before and after each intervention between the PL and CH groups in Study 2. Differences before and after each intervention were compared using a paired *t*-test. The relationships between chlorella-induced changes in any parameter were determined using Pearson’s correlation coefficient.

In Study 3, values are expressed as mean ± SD. An unpaired *t*-test was used to compare each parameter between the CON and CH groups in Study 3.

Statistical significance was set at *p* < 0.05. All statistical analyses were performed using StatView (version 5.0, SAS Institute, Tokyo, Japan), after confirming that all data were normally distributed using the Kolmogorov–Smirnov test. Cohen’s *d* was calculated by determining the mean differences in changes between two groups and dividing this value by the pooled SD. Cohen (1988) classified the effect sizes as small (*d* = 0.2), medium (*d* = 0.5), and large (*d* ≥ 0.8) [25].

## 3. Results

### 3.1. Study 1

#### Changes in Physical Characteristics and $\dot{V}$O_2max_ Before and After HIIT

**Figure 1:**
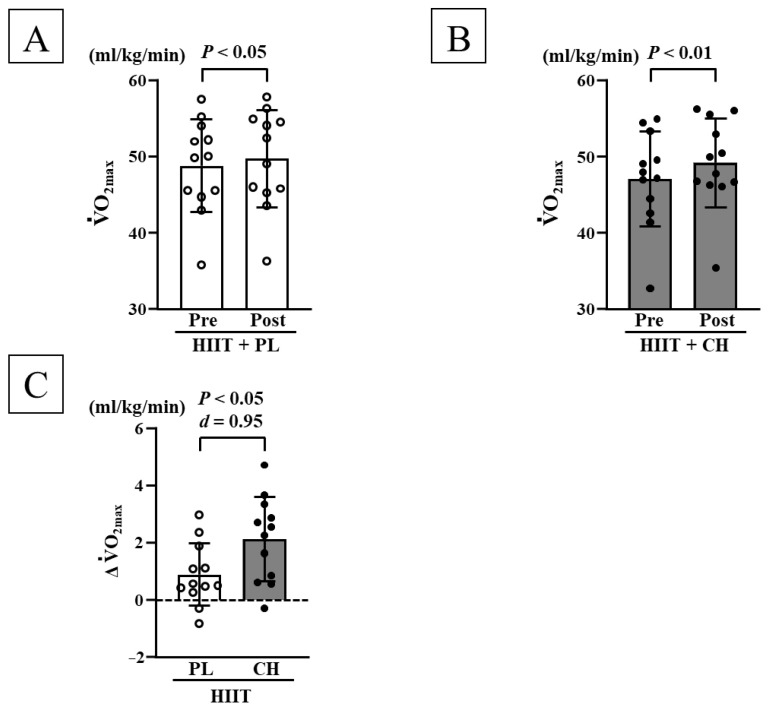
Comparison of $\dot{V}$O_2max_ before and after each intervention in the HIIT + PL (**A**) and HITT + CH (**B**) groups. Comparison of changes in $\dot{V}$O_2max_ before and after each HIIT intervention (**C**). $\dot{V}$O_2max_: maximal oxygen uptake, HIIT: high-intensity intermittent training, PL: placebo intake, CH: chlorella intake, SD: standard deviation, Δ: changes in value before and after interventions. Data are expressed as mean ± SD.

**Figure 2:**
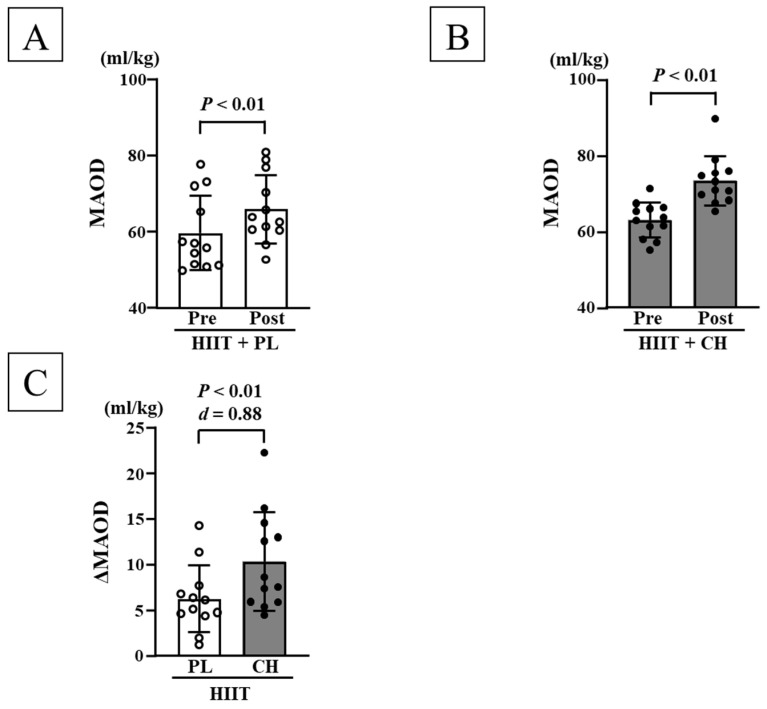
Comparison of MAOD before and after each intervention in the HIIT + PL (**A**) and HITT + CH (**B**) groups. Comparison of changes in MAOD before and after each HIIT intervention (**C**). MAOD: maximal accumulated oxygen deficit, HIIT: high-intensity intermittent training, PL: placebo intake, CH: chlorella intake, SD: standard deviation, Δ: changes in value before and after interventions. Data are expressed as mean ± SD.

Before each intervention, no significant differences were observed in body weight, body fat mass, lean body mass, resting HR, SBP, DBP, $\dot{V}$O_2max_, and MAOD among the HIIT + PL and HIIT + CH groups (Table 1). SBP decreased significantly in the HIIT + CH group after the intervention; DBP decreased significantly in both the HIIT + PL and HIIT + CH groups after each HIIT intervention (*p* < 0.05, Table 1). However, changes in SBP and DBP were not significant between the two groups. After each HIIT intervention, $\dot{V}$O_2max_ and MAOD significantly increased in both the HIIT + PL and HIIT + CH groups (*p* < 0.05; Figure 1A,B and Figure 2A,B). Additionally, changes in $\dot{V}$O_2max_ (*d* = 0.95) and MAOD (*d* = 0.88) were more significant in the HIIT + CH group than those in the HIIT + PL group (*p* < 0.05; Figure 1C and Figure 2C). No significant differences were observed before and after each intervention in body weight, body fat mass, lean body mass, and resting HR (Table 1).

### 3.2. Study 2

To clarify the mechanism of the effect of chlorella intake on aerobic capacity, we examined CO, which is a determinant of $\dot{V}$O_2max_, before and after chlorella intake.

#### 3.2.1. Changes in Physical Characteristics and $\dot{V}$O_2max_ Before and After Chlorella Intake

**Figure 3:**
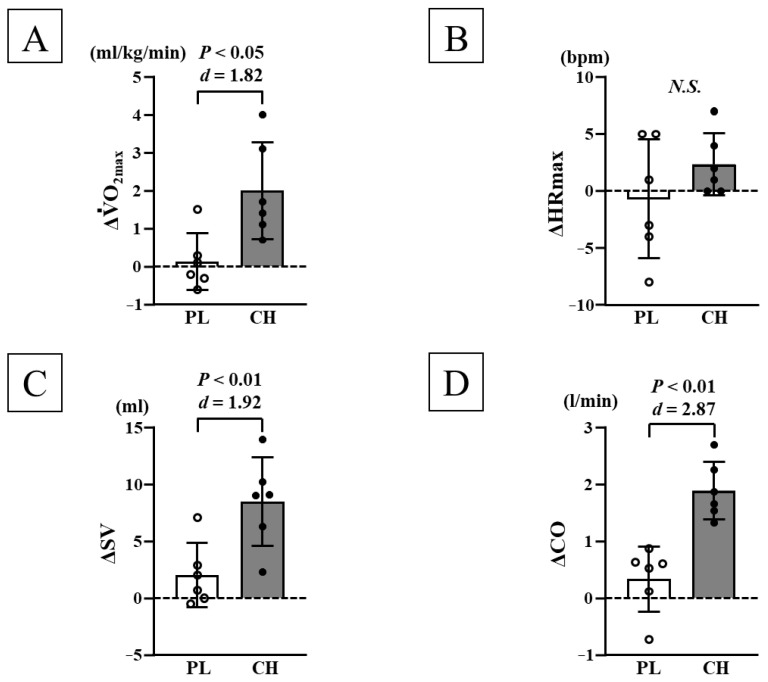
Comparison of changes in $\dot{V}$O_2max_ (**A**), HR_max_ (**B**), SV (**C**), and CO (**D**) before and after each intervention between the PL and CH groups. $\dot{V}$O_2max_: maximal oxygen uptake, HR_max_: maximal heart rate, SV: stroke volume, CO: cardiac output, PL: placebo intake, CH: chlorella intake, SD: standard deviation, Δ: changes in value before and after interventions. Data are expressed as mean ± SD.

Before each intervention, no significant differences were observed in body weight, body fat mass, lean body mass, SBP, DBP, and $\dot{V}$O_2max_ between the PL and CH groups (Table 2 and Table 3). After chlorella intake, $\dot{V}$O_2max_ increased significantly in the CH group (*p* < 0.05). Additionally, changes in $\dot{V}$O_2max_ (*d* = 1.82) were more significant in the CH group than those in the PL group (*p* < 0.05; Figure 3A). No significant differences were observed in body weight, body fat mass, lean body mass, SBP, and DBP before and after each intervention.

#### 3.2.2. Changes in Cardiac Function and Morphology Before and After Chlorella Intake

**Figure 4:**
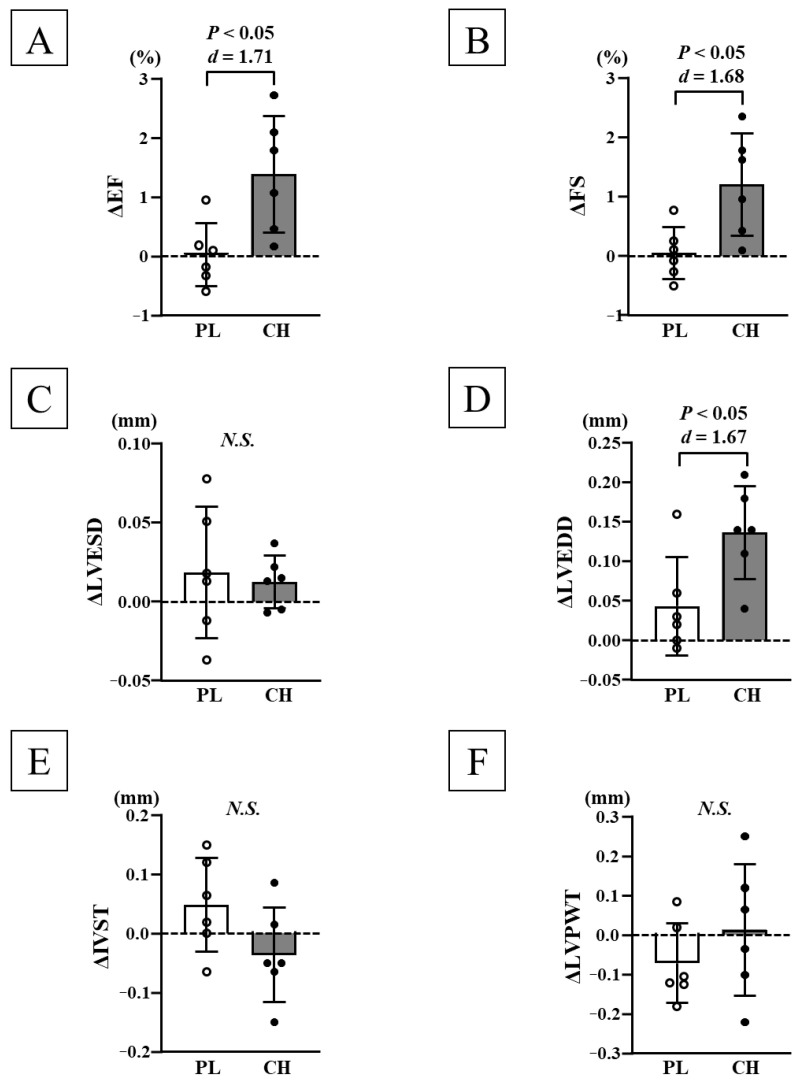
Comparison of changes in EF (**A**), FS (**B**), LVESD (**C**), LVEDD (**D**), IVST (**E**), and LVPWT (**F**) before and after each intervention between the PL and CH groups. EF: ejection fraction, FS: fractional shortening, LVESD: left ventricular end-systolic diameter, LVEDD: left ventricular end-diastolic diameter, IVST: interventricular septal thickness, LVPWT: left ventricular posterior wall thickness, PL: placebo intake, CH: chlorella intake, SD: standard deviation, Δ: changes in value before and after interventions. Data are expressed as mean ± SD.

Before each intervention, no significant differences were observed in HR, SV, CO, EF, FS, LVESD, LVEDD, IVST, and LVPWT at rest or during maximal exercise between the PL and CH groups (Table 2 and Table 3). After chlorella intake, SV, CO, EF, FS, and LVEDD during maximal exercise significantly increased in the CH group (*p* < 0.05; Figure 3C,D and Table 3). Additionally, changes in SV (*d* = 1.92), CO (*d* = 2.87), EF (*d* = 1.71), FS (*d* = 1.68), and LVEDD (*d* = 1.67) during maximal exercise were significantly higher in the CH group than those in the PL group (*p* < 0.05; Figure 3 and Figure 4 and Table 3). No significant differences were observed before and after each intervention in HR, SV, CO, EF, FS, LVESD, LVEDD, IVST, and LVPWT at rest and HR, LVESD, IVST, and LVPWT during maximal exercise (Figure 3B and Figure 4 and Table 2 and Table 3). Additionally, the LV mass index at rest did not change significantly before each intervention between the PL and CH groups; no significant differences were observed in the LV mass index at rest before and after each intervention (Table 2).

#### 3.2.3. Relationships Between Chlorella-Induced Increase in $\dot{V}$O_2max_ and Cardiac Function

Chlorella-induced increases in $\dot{V}$O_2max_ were positively correlated with increases in SV (*p* < 0.05, *r* = 0.893) and CO (*p* < 0.01, *r* = 0.948) during maximal exercise in the CH group but did not significantly correlate with changes in HR_max_.

### 3.3. Study 3

Following the results of Study 2, we examined whether the cardiac adaptations associated with the chlorella-induced increase in $\dot{V}$O_2max_ were adaptive or maladaptive by examining chlorella-induced changes in molecular markers of heart maladaptation in healthy rats.

#### 3.3.1. Comparison of Animal Characteristics

No significant differences were observed in body weight, epididymal fat mass, LV mass, gastrocnemius muscle mass, resting HR, SBP, and DBP between the CON and CH groups (Table 4).

#### 3.3.2. Comparison of Molecular Markers of Maladaptation of the Heart

**Figure 5:**
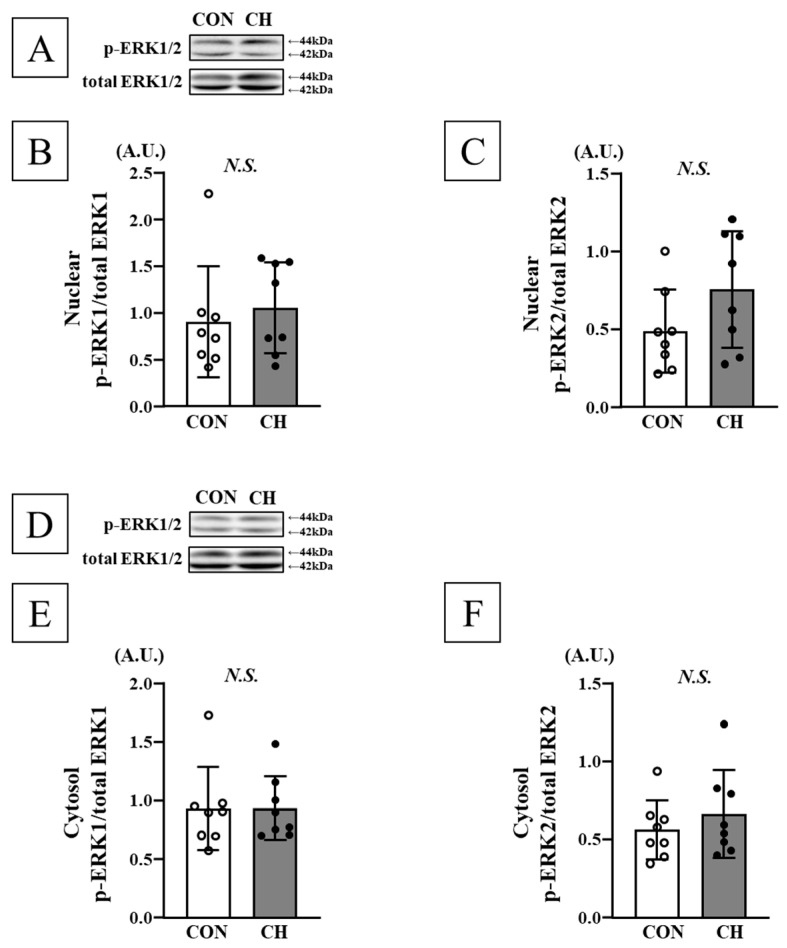
Representative Western blotting bands of nuclear ERK1/2 (**A**) and cytosol ERK1/2 (**D**). Comparison of nuclear phosphorylated ERK1 (p-ERK1; (**B**)) and phosphorylated ERK2 (p-ERK2; (**C**)), cytosol phosphorylated ERK1 (p-ERK1; (**E**)), and phosphorylated ERK2 (p-ERK2; (**F**)) in the heart between the CON and CH groups. ERK: extracellular signal-regulated kinase, A.U.: arbitrary unit, SD: standard deviation. Data are expressed as mean ± SD.

**Figure 6:**
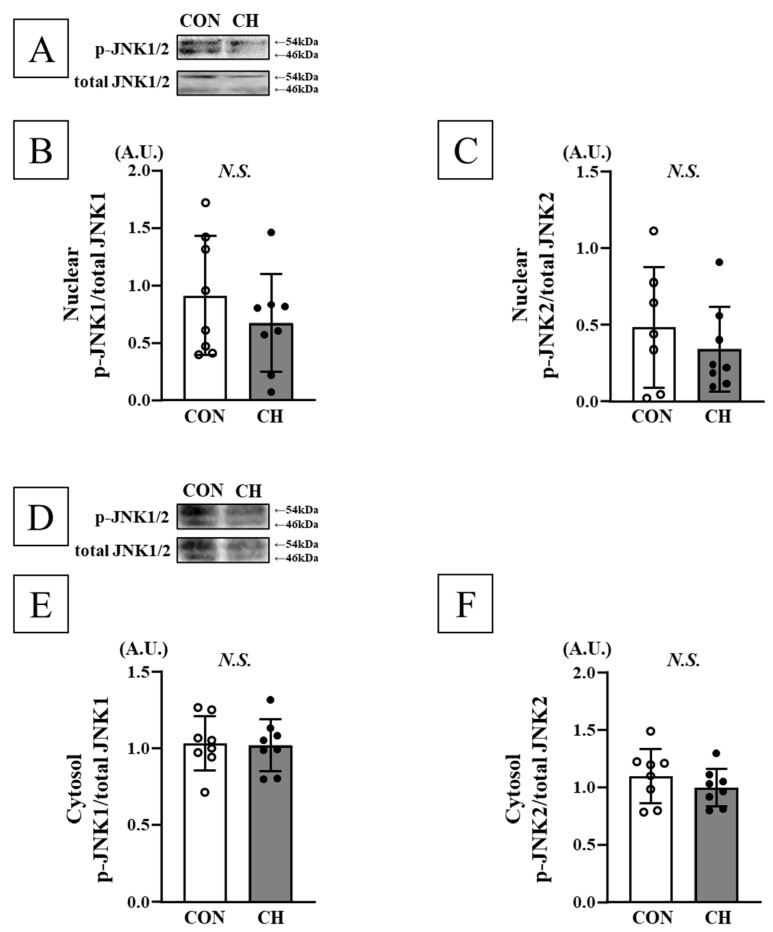
Representative Western blotting bands of nuclear JNK1/2 (**A**) and cytosol JNK1/2 (**D**). Comparison of nuclear phosphorylated JNK1 (p-JNK1; (**B**)) and phosphorylated JNK2 (p-JNK2; (**C**)), cytosol phosphorylated JNK1 (p-JNK1; (**E**)), and phosphorylated JNK2 (p-JNK2; (**F**)) in the heart between the CON and CH groups. JNK: Jun-N-terminal kinase, A.U.: arbitrary unit, SD: standard deviation. Data are expressed as mean ± SD.

**Figure 7:**
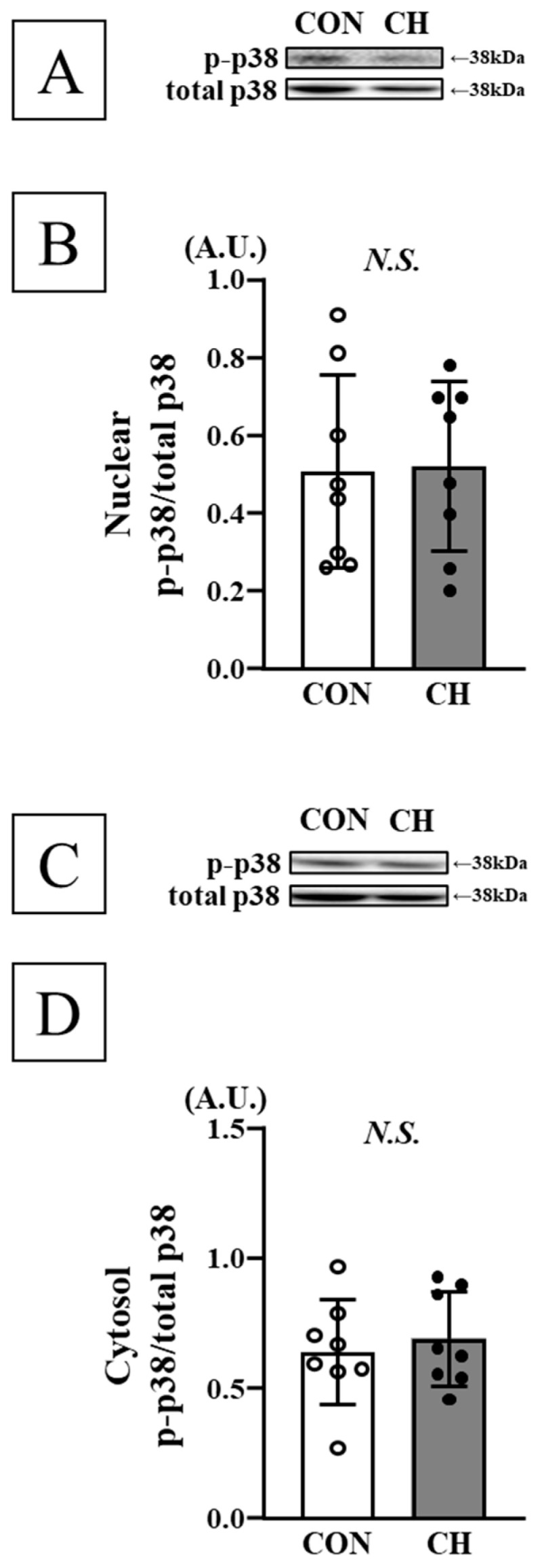
Representative Western blotting bands of nuclear p38 (**A**) and cytosol p38 (**C**). Comparison of nuclear phosphorylated p38 (p-p38; (**B**)) and cytosol phosphorylated p38 (p-p38; (**D**)) in the heart between the CON and CH groups. p38: mitogen-activated protein kinase p38, A.U.: arbitrary unit, SD: standard deviation. Data are expressed as mean ± SD.

**Figure 8:**
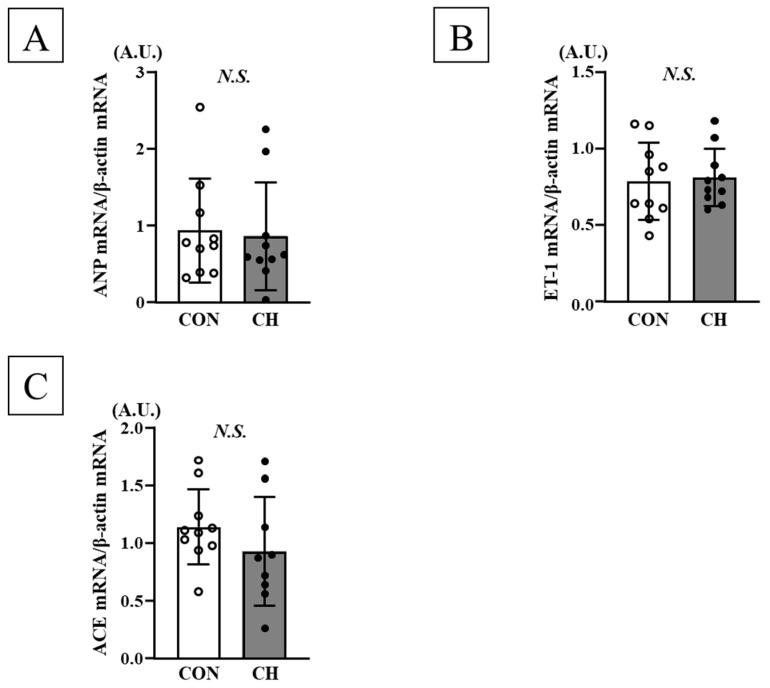
Comparison of mRNA expression levels of ANP (**A**), ET-1 (**B**), and ACE (**C**) in the heart between the CON and CH groups. ANP: atrial natriuretic peptide, ET-1: endothelin-1, ACE: angiotensin-converting enzyme, A.U.: arbitrary unit, SD: standard deviation. Data are expressed as mean ± SD.

No significant differences were observed in phosphorylation levels of ERK1/2 (p44/p42), JNK1/2 (p54/p46), and p38 in both heart nuclear and cytosolic fractions, as well as mRNA expression of ANP, ET-1, and ACE between the CON and CH groups (Figure 5, Figure 6, Figure 7 and Figure 8).

## 4. Discussion

Our main findings herein are that the combination of chlorella intake and short-term HIIT intervention additively increased aerobic and anaerobic capacities compared with short-term HIIT alone in healthy young male participants. Furthermore, in human and animal studies, chronic chlorella intake promoted cardiac function without inducing cardiac hypertrophy and did not change maladaptive molecular expression. Therefore, an increase in CO during maximal exercise was commensurate with the promotion of cardiopulmonary fitness associated with the additive effect of chlorella intake and HIIT. The mechanism behind the increase in CO involved the enhancement of cardiac function. To the best of our knowledge, this is the first study to reveal that the combination of chlorella intake and short-term HIIT additively increased exercise performance in healthy young male participants.

Chronic chlorella intake increased $\dot{V}$O_2max_ as an index of aerobic capacity, as well as SV and CO during maximal exercise as indices of cardiac function in young male participants of this study. Changes in $\dot{V}$O_2max_ before and after the intervention positively correlated with changes in SV and CO during maximal exercise but did not significantly correlate with changes in HR_max_. VO_2_ is determined using variables in the Fick equation: VO_2_ = CO (SV × HR) × arteriovenous oxygen difference (oxygen utilization in peripheral tissues such as skeletal muscles). Among several physiological factors for $\dot{V}$O_2max_, it is believed that maximal CO is the primary factor explaining individual differences in $\dot{V}$O_2max_ [26]. Thus, these data showed that the chlorella-induced increase in SV was attributed to the increase in $\dot{V}$O_2max_. Meanwhile, we did not measure arteriovenous oxygen difference in this study; however, our previous study showed that chronic chlorella intake increased muscle citrate synthase—the rate-limiting enzyme in the tricarboxylic acid cycle—activity in rats [15]. Overall, chlorella intake might additively increase CO during maximal exercise and muscle oxidative metabolic capacity, resulting in an increase in aerobic capacity in healthy young male adults. These findings, combined with previous results, offer a clearer mechanistic explanation of how chronic chlorella supplementation may enhance $\dot{V}$O_2max_.

This study showed that a 4-week chlorella intake intervention increased $\dot{V}$O_2max_, as well as SV, EF, and FS, during maximal exercise. LVESD during maximal exercise remained unchanged after the intervention, but LVEDD increased significantly. These findings may explain why the increased LV blood filling pressure increases its force of contraction, according to the Frank–Starling law of the heart. Thus, an increase in LV ejection volume in response to the increase in blood volume of LV filling may be associated with the increase in SV. In addition, the increase in LV contractility via LV hypertrophy may also be involved in the increase in SV during maximal exercise. However, no significant difference was observed in LV morphology, such as IVST and LVPWT, before and after chronic chlorella intake, both at rest and during maximal exercise. Therefore, chronic chlorella intake did not lead to cardiac hypertrophy. In fact, no significant difference in calculated LV mass index was observed before and after chlorella intake. Furthermore, in the animal experiments of this study, LV weight did not change after the 6-week chlorella intake intervention. In this study, the mechanism through which LVEDD increased could not be clarified. This study showed no enlargement in LVEDD at rest. In a previous study, short-term HIIT in untrained young men increased $\dot{V}$O_2peak_, along with CO, SV, and LV end-diastolic volume at submaximal exercise; however, it did not significantly change these parameters at rest, suggesting that increased LV filling during submaximal exercise contributes to the increase in aerobic capacity [17]. Therefore, promotions in LV distensibility and an increase in venous return during maximal exercise may be involved.

A previous study reported that classic mitogen-activated protein kinases, including ERK1/2, JNK1/2, and p38, are involved in diverse cellular processes, such as cell growth and proliferation, and apoptosis, and also induce pathological cardiac hypertrophy [27]. Additionally, mRNA expression levels of ANP, ET-1, and ACE are established molecular markers of pathological cardiac hypertrophy [28,29]. Whether the increased cardiac function during exercise induced by chronic chlorella intake, as shown in this study, is a physiological adaptation remains unclear. In our animal study, chronic chlorella intake did not induce cardiac hypertrophy or change the mRNA expression levels of ANP, ET-1, and ACE in rat hearts. Additionally, it did not alter the phosphorylation levels of ERK1/2, JNK1/2, and p38 in nuclear and cytosolic fractions. Thus, these results suggested that chronic chlorella intake may not induce cardiac maladaptation. However, the effects of chlorella on histological analysis and cardiac function assessment remain unclear. Therefore, future studies should use more direct and sensitive techniques to rule out potential maladaptive cardiac responses. These may include histological analysis to detect myocardial fibrosis or cellular remodeling using hematoxylin and eosin or Masson trichrome stain, as well as the assessment of cardiac function using echocardiographic strain imaging and pressure–volume loop analysis. In our animal study, rats (average weight: 624 g) consumed an average of 29.6 g/day of a 0.5% chlorella diet for six weeks, resulting in an intake of 237 mg/kg/day. Although this dose was lower than the human-equivalent dose, the extended supplementation period compared to the human study supports its translational relevance. However, given that these results were obtained in rodents, caution is warranted when extrapolating these findings to humans. Furthermore, the intake duration in this animal study was six weeks, and it is unclear whether a longer period of chlorella intake induces cardiac hypertrophy or maladaptive signaling. Further research is required to definitively conclude whether chlorella intake has any adverse effects on the heart.

Chlorella is a multi-supplement that contains various nutrients, such as proteins, dietary fiber, minerals, vitamins, and amino acids. Short-term branched-chain amino acid (BCAA) supplementation in trained male participants increased $\dot{V}$O_2max_ compared with placebo supplementation [30]. Additionally, a meta-analysis showed that chronic supplementation with L-arginine could increase $\dot{V}$O_2max_ in healthy people [31]. Another meta-analysis indicated that arginine supplementation improved aerobic exercise performance [32]. In recreationally active male young participants, a 10-week endurance training combined with protein supplementation increased $\dot{V}$O_2max_ compared with training combined with placebo supplementation [33]. Thus, the constituents of chlorella, such as BCAA, arginine, and protein, may contribute to enhanced aerobic capacity.

In healthy young male participants in this study, the combination of HIIT and chlorella intake additively increased MAOD as the index of anaerobic capacity. The mechanisms of increasing MAOD include (1) muscle mass, (2) muscle buffer capacity, and (3) muscle lactate formation capacity [34]. However, chlorella intake did not change lean body mass in the young male participants of this study. Thus, the unchanged muscle mass despite chlorella intake combined with HIIT may not be involved in the chlorella-induced increase in MAOD. A high correlation is observed between muscle buffering capacity and the concentration of histidine-related compounds in muscles [35]. Additionally, glutamate and citrulline are converted into arginine [36], and a meta-analysis has indicated that arginine supplementation improves anaerobic exercise performance [32]. Our previous study showed that chronic chlorella intake combined with HIIT in rats has a more pronounced effect on exercise performance; the activation of muscle lactate dehydrogenase B and an increase in the protein expression of monocarboxylate transporter 1—which transports lactate from the interstitium and plasma to the muscle—were also observed [15]. Thus, chlorella intake might accelerate muscle buffer capacity, resulting in an increase in anaerobic capacity.

This study investigated the effects of the combination of HIIT and chlorella intake on aerobic and anaerobic capacities in young male adults. In healthy young male adults, HIIT with or without chlorella intake increased $\dot{V}$O_2max_ and MAOD, whereas chlorella intake further enhanced changes in $\dot{V}$O_2max_ and MAOD by HIIT. Since the early 2000s, the number of HIIT studies has gained momentum and increased exponentially [37]. However, few studies have been reported on supplements that further enhance the HIIT-induced increase in aerobic and anaerobic capacities. These findings suggest that chronic chlorella intake may serve as a beneficial supplement to support training adaptation in individuals who are untrained. Although these current findings suggest that chronic chlorella intake may support training adaptations in untrained individuals, further research is needed to determine its potential to enhance performance in athletic populations.

This study has several important limitations. First, the 4-week wash-out period in this study might be short, since the wash-out period was at least 6 weeks in a previous study on chlorella intake [13]. No significant differences in each parameter were observed between pre-interventions; however, it remains unclear whether the effects produced by chlorella intake were eliminated. Second, the sample size in this study, particularly in Study 2, was small. Although the effect sizes for all parameters showing between-group differences were categorized as large, the small sample size increases the risk of false positive findings. As this study was partly exploratory in nature, no correction for multiple comparisons was applied. It is thus necessary to confirm these findings through replication studies with adequately large sample sizes. These *p*-values should thus be interpreted with caution. Third, since the chlorella supplement used in this study was a multi-component supplement, it is impossible to attribute the observed changes specifically to chlorella rather than to any one of its components or their synergistic effects. Fourth, although the results indicate a possible association between chlorella supplementation and improvements in aerobic performance and cardiac function, these findings should be interpreted cautiously given the lack of a comparator supplement, a non-exercise control group, a non-supplement group, and independent replication through a randomized controlled trial or crossover design.

## 5. Conclusions

Chronic chlorella intake combined with short-term HIIT further enhanced aerobic and anaerobic capacities in healthy young male adults. Notably, the chlorella-induced increase in cardiac function may be associated with increased aerobic capacity without cardiac maladaptation. Although our findings suggest a potential link between chlorella intake and cardiac functional changes, replication in studies with larger sample sizes is required, and the underlying mechanisms and long-term safety remain to be elucidated.

## Figures and Tables

**Table 1:** Comparison of subject characteristics between high-intensity intermittent training combined with placebo and chlorella groups in Study 1.

	HIIT + Placebo		*p* Value	HIIT + Chlorella		*p* Value		HIIT + Placebo	HIIT + Chlorella	*p* Value	Cohen’s *d*
	Pre	Post		Pre	Post						
Body weight (kg)	68 ± 8	67 ± 7	0.2748	68 ± 8	68 ± 8	0.8217	Δ	−0.3 ± 0.8	0.1 ± 0.9	0.6451	0.47
Body fat mass (kg)	11 ± 3	10 ± 3	0.0964	11 ± 3	11 ± 3	0.9739	Δ	−0.5 ± 0.9	0.0 ± 0.9	0.2017	0.56
Lean body mass (kg)	57 ± 6	57 ± 5	0.3460	57 ± 6	57 ± 6	0.8824	Δ	0.2 ± 0.7	0.1 ± 1.1	0.6903	0.11
HR (bpm)	60 ± 6	62 ± 11	0.5407	58 ± 7	60 ± 6	0.4421	Δ	2.2 ± 10.9	1.9 ± 7.0	0.9428	0.03
SBP (mmHg)	119 ± 10	119 ± 10	0.9757	124 ± 8	119 ± 14	0.0468	Δ	−0.6 ± 9.4	−5.3 ± 8.1	0.2200	0.54
DBP (mmHg)	66 ± 7	61 ± 6	0.0431	71 ± 8	62 ± 7	0.0030	Δ	−5.3 ± 8.0	−9.2 ± 8.4	0.2534	0.48

HR: heart rate, SBP: systolic blood pressure, DBP: diastolic blood pressure, Δ: changes in value before and after each intervention. Data are expressed as mean ± SD.

**Table 2:** Comparison of subject characteristics at rest between placebo and chlorella groups in Study 2.

	Placebo		*p* Value	Chlorella		*p* Value		Placebo	Chlorella	*p* Value	Cohen’s *d*
	Pre	Post		Pre	Post						
Body weight (kg)	64 ± 5	64 ± 5	0.5877	64 ± 5	64 ± 4	0.6833	Δ	0.18 ± 0.78	0.17 ± 0.94	0.9740	0.01
Body fat mass (kg)	8.6 ± 1.6	8.7 ± 2.1	0.7130	8.3 ± 1.3	8.5 ± 1.2	0.6047	Δ	0.17 ± 1.05	0.15 ± 0.27	0.9744	0.03
Lean body mass (kg)	55 ± 4	55 ± 4	0.9692	55 ± 4	55 ± 4	0.9727	Δ	0.02 ± 1.01	0.02 ± 1.13	0.9999	0.00
HR (bpm)	68 ± 8	65 ± 6	0.3442	67 ± 5	68 ± 9	0.9304	Δ	−2.83 ± 6.65	0.17 ± 4.45	0.3797	0.53
SBP (mmHg)	112 ± 7	116 ± 7	0.3602	118 ± 9	114 ± 7	0.2926	Δ	3.83 ± 9.33	−3.83 ± 8.00	0.1571	0.88
DBP (mmHg)	65 ± 7	61 ± 6	0.4080	63 ± 11	58 ± 3	0.3137	Δ	−3.83 ± 10.40	−4.83 ± 10.57	0.8721	0.10
SV (mL)	63 ± 2	62 ± 3	0.1634	63 ± 2	63 ± 2	0.6747	Δ	−1.83 ± 2.75	−0.28 ± 1.47	0.2489	0.70
CO (L/min)	4.3 ± 0.6	4.0 ± 0.3	0.1551	4.2 ± 0.4	4.2 ± 0.5	0.8864	Δ	−0.21 ± 0.45	−0.02 ± 0.25	0.1915	0.52
EF (%)	61 ± 3	60 ± 2	0.2433	60 ± 3	60 ± 3	0.4711	Δ	−1.13 ± 2.10	−0.61 ± 1.94	0.6670	0.26
FS (%)	33 ± 2	32 ± 1	0.2839	32 ± 2	32 ± 2	0.3174	Δ	−0.63 ± 1.29	−0.45 ± 0.98	0.7870	0.16
LVESD (mm)	3.2 ± 0.2	3.2 ± 0.2	0.5279	3.2 ± 0.2	3.3 ± 0.2	0.7803	Δ	0.02 ± 0.06	0.02 ± 0.14	0.9999	0.00
LVEDD (mm)	4.8 ± 0.2	4.7 ± 0.2	0.3790	4.8 ± 0.2	4.8 ± 0.1	0.7412	Δ	−0.03 ± 0.07	0.02 ± 0.12	0.4406	0.51
IVST (mm)	0.8 ± 0.1	0.8 ± 0.1	0.1438	0.8 ± 0.1	0.8 ± 0.1	0.2236	Δ	−0.02 ± 0.03	−0.03 ± 0.05	0.7631	0.24
LVPWT (mm)	0.9 ± 0.1	0.9 ± 0.1	0.7820	0.9 ± 0.1	0.9 ± 0.1	0.8471	Δ	0.01 ± 0.11	−0.01 ± 0.08	0.7295	0.21
LV mass index (g/m^2^)	74 ± 7	73 ± 10	0.7266	75 ± 9	74 ± 9	0.5882	Δ	−1.02 ± 6.61	−1.19 ± 5.08	0.9612	0.03

HR: heart rate, SBP: systolic blood pressure, DBP: diastolic blood pressure, SV: stroke volume, CO: cardiac output, EF: ejection fraction, FS: fractional shortening, LVESD: left ventricular end-systolic diameter, LVEDD: left ventricular end-diastolic diameter, IVST: interventricular septal thickness, LVPWT: left ventricular posterior wall thickness, LV: left ventricular, Δ: changes in value before and after each intervention. Data are expressed as mean ± SD.

**Table 3:** Comparison of subject characteristics during maximal exercise between placebo and chlorella groups in Study 2.

	Placebo		*p* Value	Chlorella		*p* Value		Placebo	Chlorella	*p* Value	Cohen’s *d*
	Pre	Post		Pre	Post						
HR (bpm)	185 ± 5	184 ± 8	0.7679	186 ± 4	189 ± 5	0.0907	Δ	−0.67 ± 2.14	2.33 ± 2.73	0.2421	0.72
$\dot{V}$O_2max_ (ml/kg/min)	43 ± 7	43 ± 7	0.2964	43 ± 6	45 ± 5	0.0404	Δ	0.13 ± 0.74	2.00 ± 1.28	0.0113	1.82
SV (mL)	119 ± 25	121 ± 25	0.1315	121 ± 26	129 ± 23	0.0032	Δ	2.03 ± 2.78	8.47 ± 3.90	0.0081	1.92
CO (L/min)	22 ± 5	22 ± 5	0.2101	23 ± 5	24 ± 5	0.0004	Δ	0.34 ± 0.57	1.89 ± 0.51	0.0006	2.87
EF (%)	70 ± 2	70 ± 2	0.8885	70 ± 3	71 ± 2	0.0175	Δ	0.03 ± 0.53	1.39 ± 0.99	0.0141	1.71
FS (%)	40 ± 2	40 ± 2	0.7711	40 ± 2	41 ± 2	0.0189	Δ	0.05 ± 0.44	1.21 ± 0.87	0.0155	1.68
LVESD (mm)	3.5 ± 0.3	3.5 ± 0.3	0.3403	3.5 ± 0.3	3.6 ± 0.3	0.2009	Δ	0.02 ± 0.04	0.01 ± 0.02	0.7495	0.19
LVEDD (mm)	5.9 ± 0.5	5.9 ± 0.5	0.1682	5.9 ± 0.5	6.0 ± 0.5	0.0034	Δ	0.04 ± 0.06	0.14 ± 0.06	0.0235	1.67
IVST (mm)	0.9 ± 0.1	1.0 ± 0.1	0.2168	0.9 ± 0.1	0.9 ± 0.1	0.3432	Δ	0.05 ± 0.08	−0.04 ± 0.08	0.0966	1.06
LVPWT (mm)	1.0 ± 0.1	0.9 ± 0.1	0.1548	0.9 ± 0.2	0.9 ± 0.1	0.8523	Δ	−0.07 ± 0.10	0.01 ± 0.17	0.3159	0.61

HR: heart rate, $\dot{V}$O_2max_: maximal oxygen uptake, SV: stroke volume, CO: cardiac output, EF: ejection fraction, FS: fractional shortening, LVESD: left ventricular end-systolic diameter, LVEDD: left ventricular end-diastolic diameter, IVST: interventricular septal thickness, LVPWT: left ventricular posterior wall thickness, Δ: changes in value before and after each intervention. Data are expressed as mean ± SD.

**Table 4:** Comparison of animal characteristics at rest between control and chlorella groups in Study 3.

	Control	Chlorella	*p* Value
Body weight (g)	587 ± 37	624 ± 48	0.0699
Epididymal fat mass (g)	7.9 ± 1.5	7.9 ± 2.8	0.9798
LV mass (g)	1.1 ± 0.1	1.2 ± 0.2	0.2403
GA muscle mass (g)	2.7 ± 0.2	2.8 ± 0.3	0.3943
HR (bpm)	285 ± 41	276 ± 15	0.7278
SBP (mmHg)	100 ± 10	94 ± 4	0.3383
DBP (mmHg)	74 ± 10	74 ± 6	0.9476

LV: left ventricular, GA: gastrocnemius, HR: heart rate, SBP: systolic blood pressure, DBP: diastolic blood pressure. Data are expressed as mean ± SD.

## Data Availability

The data presented in this study are available upon request from the corresponding author due to ethical.

## Abbreviations

The following abbreviations are used in this manuscript:

HIIT	High-intensity intermittent training
$\dot{V}$O_2max_	Maximal oxygen uptake
MAOD	Maximal accumulated oxygen deficit
HIIE	High-intensity intermittent exercise
CO	Cardiac output
LV	Left ventricular
PL	Placebo
CH	Chlorella
BDHQ	Brief-type self-administered diet history questionnaire
HR	Heart rate
SBP	Systolic blood pressure
DBP	Diastolic blood pressure
CV	Coefficients of variation
SV	Stroke volume
EF	Ejection fraction
FS	Fractional shortening
LVESD	Left ventricular end-systolic diameter
LVEDD	Left ventricular end-diastolic diameter
IVST	Interventricular septal thickness
LVPWT	Left ventricular posterior wall thickness
CSA	Cross-sectional area
ANP	Atrial natriuretic peptide
ET-1	Endothelin-1
ACE	Angiotensin-converting enzyme
RT-PCR	Reverse transcription polymerase chain reaction
ERK	Extracellular signal-regulated kinase
JNK	Jun-N-terminal kinase
PBS	Phosphate-buffered saline
SD	Standard deviation
BCAA	Branched-chain amino acid
